# What can be learned from the literature about intervals and strategies for paediatric CPR retraining of healthcare professionals? A scoping review of literature

**DOI:** 10.1016/j.resplu.2022.100319

**Published:** 2022-10-28

**Authors:** Debora Gugelmin-Almeida, Lucia Tobase, Ian Maconochie, Thatiane Polastri, Elaine Cristina Rodrigues Gesteira, Jonathan Williams

**Affiliations:** a Faculty of Health and Social Sciences, Bournemouth University, Bournemouth Gateway Building, St. Pauls Lane, Bournemouth BH8 8GP, England; b Centro Universitário São Camilo, Rua Raul Pompeia, 144, São Paulo, Brazil; c Paediatric Emergency Medicine, St Mary's Hospital, Imperial College NHS Healthcare Trust, London, UK; d Instituto do Coração do Hospital das Clínicas da Faculdade de Medicina da Universidade de São Paulo, Av. Dr. Enéas de Carvalho Aguiar, 44, São Paulo, Brazil; e Federal University of São João del Rei, Nursing Course - Child and Adolescent Health, Divinópolis, Minas Gerais, Brazil

**Keywords:** Paediatric cardiopulmonary resuscitation, Training strategies, Retraining intervals, Scoping review, Healthcare professionals, APLS, Advanced paediatric life support, ATLS, Advanced trauma life support, BLS, Basic life support, CPR, Cardiopulmonary resuscitation, EM, Emergency medical, EMS, Emergency medical services, EPALS, European paediatric advanced life support, ILCOR, International liaison committee on resuscitation, pCPR, Paediatric cardiopulmonary resuscitation, PALS, Paediatric advanced life support, PHPLS, Pre-hospital paediatric life support, PILS, Paediatric intermediate life support, RCT, Randomised controlled trial

## Abstract

**Background:**

Effective training and retraining may be key to good quality paediatric cardiopulmonary resuscitation (pCPR). PCPR skills decay within months after training, making the current retraining intervals ineffective. Establishing an effective retraining strategy is fundamental to improve quality of performance and potentially enhance patient outcomes.

**Objective:**

To investigate the intervals and strategies of formal paediatric resuscitation retraining provided to healthcare professionals, and the associated outcomes including patient outcomes, quality of performance, retention of knowledge and skills and rescuer’s confidence.

**Methods:**

This review was drafted and reported using the Preferred Reporting Items for Systematic Reviews and Meta-analysis extension for Scoping Reviews (PRISMA-ScR). PubMed, Medline, Cochrane, Embase, CINAHL Complete, ERIC and Web of Science were searched and studies addressing the PICOST question were selected.

**Results:**

The results indicate complex data due to significant heterogeneity among study findings in relation to study design, retraining strategies, outcome measures and length of intervention. Out of 4706 studies identified, 21 were included with most of them opting for monthly or more frequent retraining sessions. The length of intervention ranged from 2-minutes up to 3.5 hours, with most studies selecting shorter durations (<1h). All studies pointed to the importance of regular retraining sessions for acquisition and retention of pCPR skills.

**Conclusions:**

Brief and frequent pCPR retraining may result in more successful skill retention and consequent higher-quality performance. There is no strong evidence regarding the ideal retraining schedule however, with as little as two minutes of refresher training every month, there is the potential to increase pCPR performance and retain the skills for longer.

## Introduction

Survival from paediatric cardiac arrest is dependent on medical interventions including high quality paediatric cardiopulmonary resuscitation (pCPR).^1^, ^2^, ^3^ However, pCPR quality frequently does not meet current standards. Long interruptions and incorrect chest compression depth and rate are some of the challenges, potentially impacting positive outcomes.^4^, ^5^, ^6^

Effective training and retraining may be key to pCPR quality. Previous studies demonstrated that learners acquire CPR knowledge and skills irrespective of the method it is delivered^7^, ^8^, ^9^, ^10^, ^11^, ^12^, ^13^ however, evidence shows that CPR skills decay within weeks to months after training, demonstrating that the current retraining intervals of one or two years is ineffective.^9^, ^14^, ^15^, ^16^ This, coupled with paediatric cardiac arrest being an uncommon event, with an incidence of 8.04/100,000 for out-of-hospital cardiac arrests and around 1/1000 admissions for in-hospital cardiac arrests, further perpetuates the challenge in retaining pCPR skill.^17^, ^18^, ^19^ Current resuscitation guidelines recommend a distributed practice model for teaching and learning CPR skills, however, there is no clarity over the optimal gap between training or retraining sessions.^19^, ^20^, ^21^ It has been suggested that monthly retraining can enhance retention^16^, ^22^, ^23^ yet, this may not be feasible in clinical areas due to associated high costs, staff motivation and drop outs.^16^, ^24^ Previous reviews have explored retraining intervals for laypersons and spaced learning for resuscitation training, however these reviews did not focus on paediatric CPR. High-quality CPR has been associated with improved survival outcomes after cardiac arrest in the adult population.^25^, ^26^, ^27^ Establishing an effective retraining strategy that facilitates learning and maximizes retention of pCPR skills is fundamental to improve quality of performance and potentially enhance patient outcomes after cardiac arrest.

This scoping review aimed to provide a contemporary synthesis of the literature exploring intervals and strategies of formal paediatric resuscitation training/retraining provided to healthcare professionals, and the associated outcomes including patient-level outcomes, quality of pCPR performance, retention of knowledge and skills, and rescuer’s confidence. Since interventions such as “low dose, high frequency”^28^, ^29^ is not considered full retraining but short exposures to the skills, the term “refresher” will be used alongside retraining when appropriate.

## Methods

### Study design and protocol

In order to achieve the above stated aim, a scoping review was the preferred method. It enables to determine the scope of evidence available, provide an overview on key aspects underpinning the research area and gaps in literature to be identified.^30^

This scoping review protocol was drafted and reported using the Preferred Reporting Items for Systematic Reviews and Meta-analysis extension for Scoping Reviews (PRISMA-ScR).^31^ To the best of the authors’ knowledge, there are no existing scoping or systematic reviews exploring the same or similar research question based in the paediatric population. Ethical approval was not applicable to this study.

### Research question

The research question was based on PICOST (Population, Intervention, Control, Outcomes, Study design and Timeframe) and defined as: “What can be learned from the literature regarding strategies and intervals of paediatric CPR retraining provided to healthcare professionals in relation to patient outcomes; good quality performance; better retention of knowledge and/or skills; and/or improved rescuer’s confidence?”

P - healthcare professionals including doctors, nurses, EMS providers, Allied Health Professionals or any other healthcare professional working in any geographic location and any setting (pre-hospital, community and hospitals) undergoing formal pCPR retraining.

I - any form of formal pCPR retraining.

C - different retraining or refresher intervals.

O – patient outcome; ability to deliver effective pCPR – simulated or real; knowledge and skill improvement; retention of knowledge and skills; and rescuer’s confidence.

S - Primary studies (quantitative, qualitative and mixed-methods) including randomised controlled trials (RCTs), non-randomised controlled trials, interrupted time series, controlled before-and-after studies, observational and cohort studies were included in order to consider different aspects of measuring outcomes.

T - studies published between January 2005 and March 2022 (since the first publication of the 2005 guidelines on resuscitation by the ILCOR process, feeding scientific literature to the different Resuscitation Councils).

### Eligibility criteria

All studies addressing the PICOST question were eligible, including paediatric manikin and/or simulation; paediatric basic life support (BLS) retraining; paediatric advanced life support (PALS); European paediatric advanced life support (EPALS); advanced paediatric life support (APLS); paediatric immediate life support (PILS); pre-hospital paediatric life support (PHPLS); and advanced trauma life support (ATLS). Studies based on neonatal CPR training/ retraining; adult CPR training/retraining; healthcare students; unpublished studies and studies in a language other than English were excluded from this review.

### Search strategy

The following databases were searched by three researchers (DA, LT, TP): PubMed/Medline; Cochrane; Excerpta Medica Database (Embase); Cumulative Index to Nursing and Allied Health Literature (CINAHL) Complete and Web of Science. A pre-defined search strategy was used combining Boolean operators ‘AND’ and ‘OR’ with medical search headings and subheadings (e.g. MESH) when applicable. The search terms (Appendix 1) were drafted by the research team and revised by an experienced librarian. The reference lists from included sources were manually searched to identify any further studies not yet captured.

### Study identification and selection criteria

All articles initially identified were sent to the web-based bibliographic manager (EndNote Desktop X9) where duplicate references were removed. To increase consistency, two reviewers (DA and LT) screened the identified sources for relevance by evaluating the titles and abstracts according to the proposed eligibility criteria. Disagreement was resolved by consensus, moderated by a third reviewer (TP). If during abstract screening suitability could not be determined, further evaluation of the full text was performed, at which point, those studies that did not fit the eligibility criteria were excluded.

### Data extraction and analysis

Data from included full text sources were extracted and organised in an Excel spreadsheet using a “descriptive-analytical” method within a pre-set framework^32^ to ensure that variations between studies were uniformly captured and described. Study identification (first author, title, DOI, year of publication, geographic location); study design (context, sample size, intervention, duration); participants (profession, setting); training/retraining details (BLS, PALS, EPALS, PILS, etc.); outcomes (knowledge, confidence, ability to deliver effective CPR, skill retention, etc.); methods of assessment; results; and conclusion were summarised for further analysis.

## Results

**Fig. 1:**
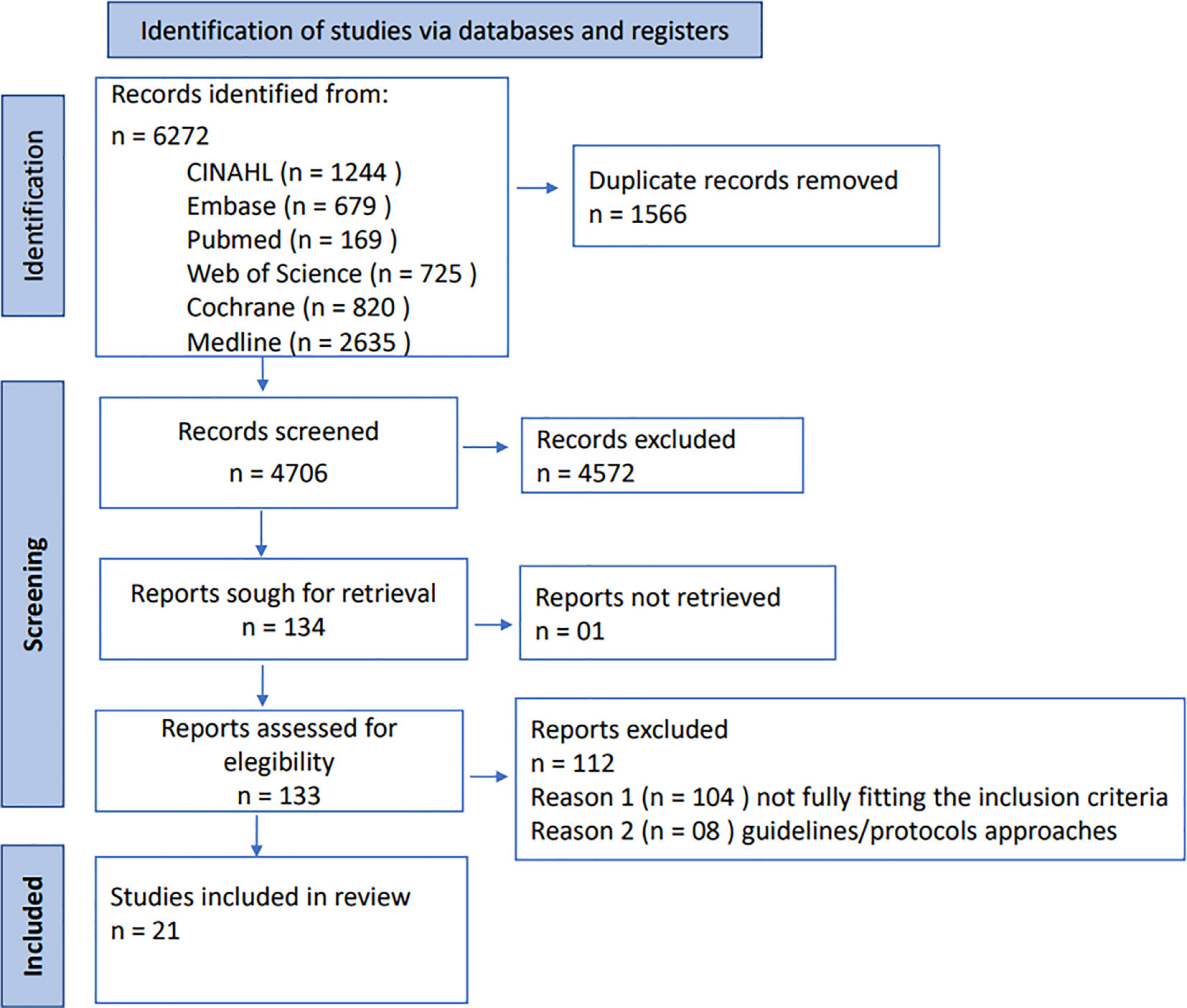
Demonstrates the PRISMA-ScR flow diagram.^31^

The initial search resulted in 6272 studies. Of those, 1566 were duplicates, resulting in 4706 titles post-deduplication. After reading titles and abstracts, 134 were selected for full text review. Of those, one could not be retrieved and 112 were discarded for not fully fulfilling the inclusion criteria, leaving 21 studies included in the analysis, as seen in Fig. 1.

### Study characteristics

Study characteristics and interventions are summarised in Table 1. Over 3000 healthcare professionals were involved including nurses, paediatric residents, emergency medicine residents, EMS providers, physicians, respiratory therapists and pharmacists.

**Table 1:** Studies characteristics, interventions and results of evidence.

*Author(year),* *Country*	*Study characteristics (design, population, sample setting)*	*Length and key points of retraining intervention*	*Outcome measures*	*Results*	*Conclusion*
Anantasit et al.^33^ (2016) Thailand	Pre-post test, 38 paediatric residents, hospital	1-hour video feedback 1 week later + test 6 weeks later	pCPR skills (depth, rate, recoil) + team-basedCPR	Skills: 50% pass test 1 / 68% (p = 0.09) pass in test 2 Team-based: 46% passed test 1 / 92% (p = 0.08) pass in test 2	Improvement of skills and team-based pCPR with reinforcement of video feedback
Andreatta et al.^34^ (2011), USA	Longitudinal, mixed-methods, 252 paediatric residents, paediatric nurses, pharmacists, hospital	Monthly mock codes. Each participant took part in at least one mock code, but more often they participated in two or more mock codes.	Confidence, skills, knowledge and patient outcome	Survival rates ↑ to approximately 50% (p < 0.001) and correlated with the increased number of mock codes (r = 0.87)	Frequent mock codes improve skills, confidence and patient outcome.
Auerbachet et al.^35^ (2011), USA	Longitudinal, prospective, interventional, 115 paediatric and emergency medicine residents, hospital	repetitive simulation (10 min scenario + 30 min video debriefing with feedback + 10 min scenario to apply feedback) vs standard simulation (10 min scenario + 30 min video debriefing with feedback). First 6 months standard simulation; last 6 months repetitive simulation	Confidence, skills, knowledge	Perceived knowledge and skills: significantly improved between repetitive and standard (p = 0.005 and p = 0.02 respectively). Perceived confidence: not significantly different (p = 0.4)	Repetitive simulation using scenario + debrief + scenario can improve perceived knowledge and perceived skills in medical residents
Biese et al.^36^ (2009), USA	pre-post, 26 paediatric and EM residents, hospital	Pre-test chest compression scenario; 20 mins screen-based high-fidelity simulated code for 4 weeks; post-test scenario	Confidence, skills, knowledge	Confidence: improved from pre to post-test (10.1 SD ± 4.9; range 0–19; p < 0.001). Overall performance: was not significant from pre-test (6.65 (±1.76) to post-test (7.04 (±1.37); p = 0.58)	Frequent screen‐based simulation may be a useful adjunct in educating residents to manage paediatric resuscitations by enhancing knowledge, confidence, and some skills.
Bishop et al.^37^ (2018), USA	prospective, interventional, 62 PICU nurses, hospital	Monthly training with RTF for 2 minutes. Nurses with 3 or more training sessions before their final data collection = experienced trainees Nurses with 2 or fewer training sessions = novice trainees.	Target of high-quality CPR for more than 70% based on depth and rate	As the number of training sessions increased, the percentage of CPR in the target range also increased, with less variability in performance. 29% with no training 46% after 1 session, 54% after 2 sessions, 68% after 3 sessions, 74% after 4 sessions (p = 0.001). Median percentage of time in the target area was 68% (interquartile range [IQR], 64–72) among the experienced trainees and 48% (IQR, 43–59) among novice trainees; p = 0.002).	Repeated short refresher with RTF significantly increase performance
Braun et al.^38^ (2015), USA	RCT, 42 paediatric residents, hospital	Baseline performance; Repeated scenario as needed until mastery-level performance was achieved (1 h to 2 h to achieve mastery); Retest 2, 4, or 6 months later	Retention of mastery-level performance	Percentage of residents maintaining mastery-level performance showed a significant linear decline (p = 0.039), with a drop at each retesting interval. 92% retained mastery at 2 months; 71% at 4 months, 56% at 6 months.	Significant improvements in resuscitation performance after a single simulation-based mastery learning session. However, performance declined over time. Relatively frequent refresher training is needed after a single simulation-based mastery learning session.
Chang et al.^39^ (2019), UK	RCT, 920 healthcare professionals expected to perform CPR, hospital	Pre-test 2 min infant CPR with RTF; practice as many times as wanted for 2 minutes during 8 months. 4 months control (no display on leaderboard) and 4 months intervention (display results on leaderboard)	Leaderboard scores, frequency of practice, CPR performance	2.14 practice episodes per participant during the control phase; 1.94 episodes per participant during the intervention (just a few participants practised more than once). No significant change in performance.	No lasting improvements in either frequency of CPR practice or CPR performance scores in the presence of a leaderboard.
Ciurzynski et al.^40^ (2017), USA	Pre-post, 21 nurses, hospital	Pre-test questionnaire; 8–12 minutes, 2 rescuers simulated CPR with RTF; switch roles; (if an overall CPR performance score of 80% was not achieved, repeat CPR with RTF); debrief; post-test questionnaire and refresher at 6 months	Knowledge, CPR performance, comfort with emergency response	Knowledge: significantly improved (p = 0.001); knowledge was not retained at 6 months (97, SD = 6) and (85, SD = 11), p = 0.001; Comfort: significantly higher (p = 0.004); Skills: improved at 6 months but not significantly	A personalised refresher simulation every 6 months is recommended
Donogue et al.^41^ (2021), USA	Observational, 253 physicians, nurses, paramedics, EM technicians, hospital	Baseline assessment; CPR self-directed skill training every 3 months where participants had to pass with a score over 75%. If not, RTF until pass; AND real-life events with chest compression monitor and videorecording.	Chest compression within guidelines for depth and rate	Statistically significant improvement for infant CPR (91.5 and 95.0p = 0.03) and paediatric CPR (84.3 and 96.2p < 0.001) between the first and last quarters of the study period. Independent association between a greater number of sessions and adherence to guidelines for rate. No improvement in chest compression depth during actual CPR events.	High-frequency, brief CPR training led to consistently increased performance of high-quality CPR in ongoing training sessions.
Garcia-Jorda et al.^42^ (2019), Canada	Observational, 194 physicians, nurses and medical residents, hospital	2 min blinded CPR; up to 3x 2 min trials to achieve 90% with RTF; 2x monthly simulated CPR; debrief. Skill retention measured according to availability: block 1 (1–3 months); block 2 (3–6 months); block 3 (over 6 months)	CPR performance (depth, rate and recoil); Excellent CPR (retention above 90% for each metric or combined; Retention of skills	Rate: 73% trial 1; 91% trial 2; 92% trial 3 Depth and recoil: 100% trials 1, 2 and 3 Excellent CPR: 29% trial 1; 46% trial 2; 48% trial 3 Retention: Rate: baseline (97%); 1–3 months (66%); 3–6 months (69%); > 6 months (78%) Depth: baseline (99%); 1–3 months (96%); 3–6 months (94%); > 6 months (95%) Recoil: baseline (99%); 1–3 months (98%); 3–6 months (98%); > 6 months (98%)	Short rolling refresher trainings should be implemented regularly
Hunt et al.^43^ (2018), USA	Observational, 241 paediatric residents, hospital	Weekly 90 mins real cardiac arrest performance debrief	Excellent CPR (proportion of cycles compliant for depth, rate and chest compression fraction)	Excellent CPR 2013: 19.9 (6.9, 32.9); 2014: 41.8 (30.5, 53.0); 2015 = 44.3 (35.3, 53.3); p = 0.04 3.2 increase in the odds of excellent CPR from 2013 to 2015 [95% (1.3–8.1), P = 0.01]	Post‐event debriefing program + RTF is associated with measurable improvements in actual resuscitation performance
** Jani et al.^44^ (2019), USA	RCT, 24 paediatric residents, hospital	**Distributed practice** MCQ pre-course; skills stations and debrief at month 4 (intervention); MCQ and skills at month 8	Skill retention; knowledge	Intervention group performed better at 8 months than control (p = 0.04). But skills decayed from baseline to 4 months, and 4 months to 8 months; MCQ scores: significant differences from pre to follow up (p < 0.001)	Simulation-based curricula with deliberate practice and debriefing provide a potential pathway for safeguarding against the decay of resuscitation skills
** Kurosawa et al.^45^ (2014), USA/Japan	RCT, 40 PICU nurses and respiratory therapists, hospital	**Distributed practice** six 30-minute (reconstructed PALS) delivered over 6 months	Skill; behavioural performance	Skill: pre= (16.3 ± 4.1 post, 22.4 ± 3.9; p < 0.001). Behavioural performance: pre= (33.3 ± 4.5 vs post, 35.9 ± 5.0; p = 0.008)	PALS-reconstructed training is feasible and more effective than standard PALS for skill performance.
** Lin et al.^46^ (2018), Canada	RCT, 87 paediatric healthcare providers, hospital	**Distributed practice** Group 1: distributed CPR training with RTF at least once month (no max practice number) Group 2: traditional CPR training Retest all at 3-month and 12-month	Performance and retention based on depth, rate and recoil; excellent CPR (90% for depth, rate and recoil)	Group 1: 85% practised monthly; Performance: group 1 significantly improved at 3 months (depth, p < 0.001; rate p < 0.001; and recoil, p < 0.001) and performance was retained at 12-months. Group 2 did not improve at 3-months for compression depth and recoil decayed significantly (p = 0.030). Retention: at 12-month follow up, group 1 improved significantly compared to group 2 for proportion of excellent CPR: (19.5% vs 71.7%, p < 0.001).	Distributed short practice model with RTF improves the quality of CPR and the long-term skill retention
Mariani et al.^47^ (2019), USA	Pre- and post-test, 18 paediatric nurses, hospital	Control group: baseline knowledge assessment + self-confidence survey; mock code at 9-months; knowledge assessment and self-confidence survey at 11-months; Intervention group: baseline knowledge assessment and self-confidence survey; simulation with debriefing at months 1, 5 and 9; knowledge assessment and self-confidence survey at month 11.	knowledge, skills, self-confidence	No statistically significant difference between groups at baseline; Statistically significant difference in the post-test scores (p = 0.016) with the intervention group scoring higher than the control group. No statistically significant differences in self-confidence or final scenario between the groups.	Repeated paediatric mock code simulations with structured debriefing can be an effective method to educate CPR skills.
Niles et al.^22^ (2009), USA	Prospective, observational, 420 nurses, physicians, respiratory therapists, hospital	Refresher sessions for less than 5 minutes. Group 1: less than 2 refreshers a month; Group 2: more than 2 refreshers a month.	Time to achieve good quality CPR based on rate, depth and recoil	Time to achieve good quality CPR: refreshed ≥ 2 times/month (median 21 s, IQR: 15.75–30 s) was significantly less than those that refreshed < 2 times/month (median 67 s, IQR: 41.5–84 s), (p < 0.001)	“Rolling Refresher” bedside CPR skill training approach using “just-in-time” and “just-in-place” education is effective and well received by PICU staff.
Ojha et al.^48^ (2014), Australia	Prospective, observational, 54 doctors and nurses, hospital	Observation of 6 scenarios (10 min + 5 min debrief) fortnightly	knowledge scores; self-reported confidence levels for rate, depth, recoil	Statistically significant difference in pre (69%) and post (81%) MCQ scores (p = 0.003). Improved self-reported confidence levels at 6 months compared with baseline (72% and 35%) p < 0.001.	Repeated observation of brief scenarios has significantly improved the knowledge and confidence of HCPs.
** Patocka et al.^49^ (2019), Canada	RCT, 49 EMS providers, pre-hospital	**Distributed practice** Spaced PALS 3.5 h weekly over 1 month vs traditional PALS (2x 7 h)	Retention of skills; knowledge; self-efficacy	Skills improved immediately following the training in both traditional (pre, 1.3 ± 0.7 vs post, 3.1 ± 0.1.2; p < 0.0001) (Cohen’s d = 1.8) and spaced groups (pre, 1.6 ± 1.1 vs post, 2.9 ± 1.2; p = 0.0001) (Cohen’s d = 1.1). 3-months skills: remained significantly improved from baseline in both the traditional (pre, 1.3 ± 0.7 vs post-3-months, 2.5 ± 1.5; p = 0.01) (Cohen’s d = 1) and spaced groups (pre, 1.6 ± 1.1 vs post-3-months, 2.5 ± 1.3; p = 0.01) (Cohen’s d = 0.7); MCQ: no decay for spaced group at 3-months (post training, 30.3 + 0.5 vs post-3-months 29.7 ± 0.5; p = 0.39); but statistically significant decay in the traditional group (post training, 31.1 ± 0.5 vs post-3-months 29.6 ± 0.5; p = 0.04) (Cohen’s d = 0.6). Self-efficacy scores: improved immediately following the course in both groups; however 3-months post-course only the spaced group’s scores remained significantly above baseline scores	Resuscitation training should be replaced or supplemented with frequent, spaced practice.
Sand et al.^50^ (2021), Norwich	RCT, 119 nurses, hospital	Group 1 (SS): 2-min skills station (SS) with retest at 2 and 8 months; Group 2 (SS-R): 2-min skills station + retraining at 2 months and retest at 2 and 8 months; Group 3 (IT): 2 h instructor training with retest at 2 and 8 months	CPR quality based on rate, depth, recoil, proportion of correct compression and ventilation	SS performed a higher proportion of correct ventilations compared to IT (71% and 54% respectively, N = 63, p = 0.04). The remaining CPR quality parameters were statistically similar between the two groups. SS-R had deeper compressions at 8 months (3.4 mm (7.6%, p = 0.02) and 2.8 mm 6.3%, p = 0.02). No additional benefit of retraining at 2 months could be seen at the final test. Overall test pass was approximately 17% at final evaluation for both SS-R and SS groups as compared to 7% for the IL group at 8 months, although this was not statistically significant.	CPR skill station led to similar CPR skill performance at 2 and 8 months compared to instructor led training.
Sutton et al.^28^ (2011), USA	RCT, 89 paediatric in-hospital care providers with BLS training, hospital	(1) instructor-only training; (2) automated defibrillator feedback only; (3) instructor combined with automated feedback; (4) control (no structured training). Session: baseline evaluation (60 seconds), booster training (120 seconds), and a post-training evaluation (60 seconds). 20 min in total (5 min each session x4) Control was just baseline evaluation Time: 0, 1, 3, and 6 months after training	Retention based on depth, rate, leaning, pauses	Retention of CPR skills was 2.3 times (95% CI: 1.1–4.5; p = 0.02) more likely after 2 training and 2.9 times (95% CI: 1.4–6.2; p = 0.005) more likely after 3 training sessions. The automated defibrillator feedback only group had lower retention rates compared with the instructor-only training group (odds ratio: 0.41 [95% CI: 0.17–0.97]; p = 0.043).	Brief and frequent bedside booster CPR training improves CPR skill retention.
Tofil et al.^51^ (2009), USA	Pre-post, 85 paediatric residents, hospital	20 codes over 1 year: 10–15 min scenario + 5–10 min debrief	Perception of skill; confidence	Perception skill and confidence indexes improved (p < 0.0001).	Paediatric mock codes can improve resident confidence and self-assessment of their resuscitation skills.

Notes. ** Use of distributed practice as part of learning strategy prior to retraining intervention; EM: emergency medical.

The geographical areas consisted of Thailand,^33^ USA,^22^, ^28^, ^34^, ^35^, ^36^, ^37^, ^38^, ^40^, ^41^, ^43^, ^44^, ^47^, ^51^ Japan/USA,^45^ UK,^39^ Canada,^42^, ^46^, ^49^ Australia^48^ and Norway.^50^ The methodology varied significantly and included pre/post-test,^33^, ^36^, ^40^, ^47^, ^51^ mixed-methods,^34^ interventional studies,^35^, ^37^ RCTs^28^, ^38^, ^39^, ^44^, ^45^, ^46^, ^49^, ^50^ and observational studies.^22^, ^41^, ^42^, ^43^, ^48^

Different training strategies were observed, with the majority of studies using PALS or BLS as initial training models. Most of the interventions (14) were team-based training^33^, ^34^, ^35^, ^36^, ^40^, ^43^, ^44^, ^45^, ^46^, ^47^, ^48^, ^49^, ^50^, ^51^ but of those, 12 studies included cognitive and/or psychomotor skill practice on individual level.^33^, ^34^, ^35^, ^36^, ^40^, ^43^, ^44^, ^45^, ^46^, ^47^, ^49^, ^50^ Interventions included the traditional instructor-based training;^28^, ^33^, ^45^, ^49^ simulation-based mock code program;^34^, ^47^, ^51^ simulation only;^38^ simulation with debriefing;^35^, ^44^, ^48^ high-fidelity simulation;^47^ feedback of performance;^33^, ^43^ refresher sessions;^22^, ^41^ training with real-time feedback;^28^, ^37^, ^39^, ^40^, ^41^, ^42^, ^50^ and distributed practice.^44^, ^45^, ^46^, ^49^ As some studies used distributed practice as part of their learning strategies prior to retraining interventions,^44^, ^45^, ^46^, ^49^ the authors highlighted these studies in the results table (Table 1), as the concept of distributed practice may have a positive effect on performance and retention of skills.^9^ Based on training strategies, there was an improvement in outcome measures for most of the study designs.^22^, ^28^, ^33^, ^34^, ^35^, ^36^, ^37^, ^42^, ^43^, ^44^, ^45^, ^46^, ^47^, ^48^, ^49^, ^51^ Three studies did not see significant or lasting improvement when using real-time feedback^39^, ^40^, ^50^ and one study using simulation only as a training strategy resulted in improvement but with decline over time.^38^

Outcome measures have also differed between studies and comprised of patient outcomes;^34^ pCPR skill metrics (depth, rate, recoil, chest compression fraction, pauses);^28^, ^33^, ^34^, ^35^, ^36^, ^37^, ^39^, ^40^, ^41^, ^42^, ^43^, ^44^, ^45^, ^46^, ^47^, ^50^ retention of pCPR skills;^28^, ^38^, ^42^, ^44^, ^46^, ^49^ knowledge;^34^, ^35^, ^36^, ^40^, ^44^, ^47^, ^48^, ^49^, ^51^ behavioural performance;^45^ confidence;^34^, ^35^, ^36^, ^40^, ^47^, ^48^, ^49^, ^51^ time to achieve good quality pCPR;^22^ and frequency of practice.^39^

Methods to assess outcome measures included hospital record for cardiac arrest survival rates;^34^ video recording;^34^, ^35^, ^41^, ^49^ Likert scale;^34^, ^35^, ^40^ questionnaires;^36^, ^40^, ^46^, ^47^, ^48^, ^49^ automated skill evaluation;^22^, ^28^, ^33^, ^37^, ^39^, ^40^, ^41^, ^42^, ^43^, ^45^, ^46^, ^50^, ^51^ observable scoring metrics;^28^, ^38^, ^47^, ^49^, ^51^ written assessment;^44^, ^49^ and visual analogue scale.^49^

### Retraining intervals and number of retraining sessions

The studies selected for this review used different timeframes for the first reinforcement session after initial pCPR training. Two studies performed the interventions straight after training^28^, ^40^ and one study requested participants to return one hour after initial training.^38^ Four studies brought their participants back one week after training^33^, ^36^, ^43^, ^49^ and three studies two weeks after training.^22^, ^42^, ^48^ Most study designs had the first refresher intervention at one month after training;^34^, ^37^, ^39^, ^45^, ^46^, ^51^ one study after two months;^50^ one study after three months^41^ and two studies brought their participants back after four months.^44^, ^47^

The number of refresher interventions throughout the study periods also varied considerably, with most studies doing monthly refresher sessions.^34^, ^35^, ^37^, ^45^, ^46^, ^51^ Five studies offered just one refresher session after initial training;^33^, ^38^, ^40^, ^44^, ^50^ three studies had weekly re-exposure of pCPR skills^36^, ^43^, ^49^ and further three studies fortnightly.^22^, ^42^, ^48^ Two studies offered refresher sessions every-two or three months^28^, ^41^ and one study every-four months.^47^ Interestingly, one study offered participants unlimited refresher opportunities, however, it resulted in no lasting improvement of the outcome measures.^39^ Based on the number of refresher interventions throughout the study periods, it was noted that the outcome measures had similar positive results for most variables in each study included, apart from five of six studies that offered just one refresher intervention after training^38^, ^40^, ^44^, ^50^

### Length of intervention during retraining sessions

The length of intervention in each retraining session varied considerably, ranging from 2-minutes up to 3.5 hours. For easier identification and analysis, the studies were grouped as short duration (<1h)^22^, ^28^, ^34^, ^35^, ^36^, ^37^, ^36^, ^37^, ^38^, ^39^, ^40^, ^44^, ^45^, ^46^, ^47^, ^48^, ^50^, ^51^ and long duration (≥1h).^33^, ^38^, ^43^, ^49^ Four studies^34^, ^41^, ^44^, ^47^ did not specify the length of retraining intervention, however, the interventions consisted of mock codes with/without debrief^34^, ^44^, ^47^ and self-directed short skill training^41^ and for this reason, the studies were added into the “short duration” interventions.

### Short duration (retraining interventions lasting < 1 h)

Studies investigating interventions using short durations per session were the majority (17 studies). The length of intervention ranged between two to 40 minutes and the outcome measures included patient outcome;^34^ knowledge;^34^, ^35^, ^36^, ^40^, ^44^, ^47^, ^48^, ^51^ skills/retention;^28^, ^34^, ^35^, ^36^, ^37^, ^40^, ^41^, ^42^, ^44^, ^45^, ^46^, ^47^, ^48^, ^50^ confidence;^34^, ^35^, ^36^, ^40^, ^47^, ^48^, ^51^ frequency of practice^39^ and time to achieve good quality pCPR.^22^

Patient outcome was analysed in one study^34^ and resulted in improved survival rate. Of the 14 studies analysing pCPR skill metrics, seven resulted in improvement of the skills after the retraining session.^35^, ^37^, ^41^, ^42^, ^45^, ^46^, ^50^ Participants’ knowledge was assessed in eight studies and improvement seen in seven of them.^34^, ^35^, ^40^, ^44^, ^47^, ^48^, ^51^ Retention of pCPR skills was observed in four of the six articles.^28^, ^40^, ^42^, ^46^ Seven articles analysed rescuer’s confidence with five of those resulting in improvement.^34^, ^36^, ^40^, ^48^, ^51^ Lastly, just one study in this group did not find a significant difference in outcomes at follow up.^39^ Results from interventions are described in Table 1.

### Long duration (retraining interventions lasting ≥ 1 h)

Four studies used longer lengths of interventions when retraining participants, with two of them lasting 1 h,^33^, ^38^ one lasting 90 minutes^43^ and one lasting 3.5 h.^49^ The outcome measures included skills;^33^, ^43^, ^49^ knowledge;^49^ retention of skills;^38^, ^49^ and confidence.^49^ Only one study^38^ did not improve the outcome measure at follow up. Results from interventions are described in Table 1.

## Discussion

This research has broadly and systematically identified and analysed studies relevant to retraining schedules of paediatric resuscitation skills for healthcare professionals. The International Liaison Committee on Resuscitation (ILCOR) has stated that regular pCPR skills updates are important however, the ideal retraining interval has yet to be established as evidence is limited in both quantity and quality.^19^ This review aimed to contribute to current knowledge for a further understanding of the challenges of pCPR learning and retention, from which future research can be planned. Although the researchers believe that this review will be the first step to map the gaps in knowledge and the consensus around pCPR retraining intervals for a broad overview of evidence, future research should investigate existing knowledge gaps associated with paediatric CPR training and retraining. Cost effectiveness is an important aspect that requires further exploration, particularly when retraining or refresher is delivered during clinical practice. Little is known around the impact of training or retraining strategies on patient-level outcomes as previous studies investigating variables such as survival to hospital discharge or neurological outcome are limited in both quality and quantity.^19^ Another aspect that warrants further research is whether the same retraining interval is applicable throughout the career, and whether this should vary according to the skills being trained (e.g. chest compressions, ventilation, intubation). Furthermore, assessment of the optimal strategies to team-based training, non-technical skills and leadership skills would be an important addition to the current evidence to paediatric CPR training and retraining.

In this review exploring retraining schedules of paediatric resuscitation skills for healthcare professionals, although the inclusion and exclusion criteria were well defined, the findings reveal complex data with studies that do not fit precisely into the categories. Despite internal quality assurance and transparency in reporting, identifying the time schedule and length of interventions was not simple, due to differing study methods and interventions. Additionally, the lack of clarity in some studies regarding the length of retraining interventions, made it more difficult to analyse the results.

The included studies demonstrated that the initial acquisition of pCPR skills is similar, irrespectively of the training model used. Different strategies were observed, including the traditional instructor-based training;^22^, ^28^, ^33^, ^41^, ^45^, ^49^ simulation-based mock code program;^34^, ^47^, ^51^ simulation;^32^, ^36^, ^38^, ^44^, ^48^ distributed practice;^45^, ^46^, ^48^ and training with real-time feedback.^28^, ^33^, ^37^, ^39^, ^40^, ^42^, ^43^, ^50^ Although the learning outcomes were similar, in the sense that learners acquired the skills, a better understanding of the impact of instructional designs on learning outcomes would enable researchers to design training programmes that translate into effective performance during real resuscitation attempts.^20^ This is supported by evidence from recent reviews exploring training strategies to improve CPR performance and patient outcomes.^52^, ^53^ Lauridsen and colleagues^52^ explored in their scoping review, different types of CPR training for healthcare professionals. They included aspects such as training approaches (e-learning, instructor-based, virtual reality, simulation, gamified learning); training duration and intervals; equipment and feedback (manikins, feedback devices, debriefing). The authors concluded that there is growing evidence advocating online learning and low-dose, high frequency CPR training to acquire CPR knowledge; the use of feedback devices to improve the quality of CPR skills; and team-based simulation with debriefing to enhance team performance manging a cardiac arrest. Additionally, Yeung et al.^53^ conducted a systematic review comparing spaced learning with traditional massed learning to investigate whether spaced learning strategy improves educational and clinical outcomes. Although no conclusion can be made regarding patient outcomes, the results from their review suggested that spaced learning is more effective than massed learning for performance of CPR skill after training and at follow up. Our current scoping review adds to this body of evidence but provides additional contribution through the specific focus on paediatric CPR. Similar findings were determined suggesting brief and frequent practice enhances learning of paediatric CPR.

The best training and retraining designs should be tailored to specific learning objectives, learner type and needs, or context of learning. There are recommendations related to the use of deliberate practice, mastery learning, booster training, in situ education, real-time feedback and other strategies for training and retraining.^9^, ^20^ However, despite initial learning acquisition straight after training, CPR skills normally decay within weeks after initial training.^16^, ^54^ Although some providers retain CPR skills through recurring exposure to managing cardiac arrests as part of their clinical practice,^55^ most paediatric providers go through long periods of clinical practice without performing pCPR due to the low incidence of cardiac arrest in this population.^18^ Therefore, it becomes very important to establish retraining intervals to ensure that pCPR skills are maintained for longer.

In this review, all studies pointed to the importance of regular retraining sessions to the retention of pCPR skills. Although there is no consensus on the optimal interval, most of the studies opted for refresher sessions on a monthly basis^34^, ^35^, ^37^, ^45^, ^46^ or more frequently (weekly, fortnightly).^22^, ^36^, ^37^, ^39^, ^42^, ^43^, ^48^, ^49^, ^51^ Their results indicate that frequent sessions enhance simulated pCPR outcomes with significant improvement in the outcome measures (survival rate, skills, knowledge, retention, and/or rescuer’s confidence). Conversely, the studies with less than monthly refresher sessions^28^, ^33^, ^38^, ^40^, ^41^, ^44^, ^47^, ^50^ suggest non-improvement, or improvement in one aspect but not others, or decline of skills at follow up, with one study suggesting that retention of skills was more likely with more refresher sessions.^28^ The length of intervention in each retraining session varied considerably between the studies, ranging from two minutes up to 3.5 hours. Nevertheless, it was demonstrated that, with as little as two minutes of refresher session every month, there is the potential to increase pCPR performance and retain the skills for longer. However, this cannot be directly associated with patient-level outcomes.

Although regular updates are beneficial to retention of skills, frequent retraining sessions can be associated with high dropout rates.^16^ This, aligned with significant increased costs of repeated retraining and backfilling of staff in clinical areas, may affect the viability of a high frequency training in practice. Therefore, an effective balance between retraining and sustainability has yet to be established. To reduce the burden and costs of moving practitioners away from clinical areas for lengthy pCPR retraining, short duration of intervention (as established by the current study) was adopted by most researchers included in this review.^22^, ^28^, ^34^, ^35^, ^36^, ^37^, ^39^, ^40^, ^41^, ^42^, ^44^, ^45^, ^46^, ^47^, ^48^, ^50^, ^51^ This is in alignment with other research exploring the benefits of low-dose and high-frequency or distributed practice, suggesting that retention of CPR skills may be optimised and costs reduced, by training sessions with short interventions.^14^, ^23^, ^28^, ^46^ Lin and colleagues^56^ investigated cost-effectiveness and outcomes of distributed paediatric CPR training using real-time feedback. Their results suggest that this strategy is associated with improved CPR quality and decreased training costs when compared with conventional annual mass CPR training. Despite this, further research is needed to investigate whether distributed practice affects the need for subsequent retraining intervals.

The use of feedback devices during retraining was observed in many studies.^28^, ^37^, ^39^, ^40^, ^41^, ^42^, ^43^, ^46^, ^49^, ^50^ Previous research has established the benefits of such devices during adult and paediatric CPR training.^57^ This was also observed in this review, with the majority achieving an improvement in outcome measures. Nevertheless, although the use of real-time feedback devices has been associated with enhanced performance during CPR training, there are conflicting interpretations regarding its efficacy during real-life resuscitation attempts.^58^ Therefore, whilst it may be intuitive to presume that real-time feedback devices can improve patient outcome, this is yet to be established.

This review has demonstrated that brief and frequent pCPR retraining using simulation and additional tools such as real-time feedback devices can potentially develop skills, knowledge and confidence in pCPR performance. It is also noted that increasing the frequency of retraining sessions may result in a more successful skill retention and consequent higher-quality performance. Despite this, there is no strong evidence regarding the ideal retraining schedule. It is suggested that a more nuanced approach to pCPR retraining, based on specific learning objectives, context and learners’ needs and/or performance is recommended in an attempt to maximise skill retention and improve pCPR performance.

## Limitations

This study has some limitations. First, potential biases were not systematically addressed like in a systematic review. Second, the heterogeneity among study design, retraining strategies, outcome measures and length of intervention, may impact the interpretation and synthesis of the results. Third, most studies were performed in a simulated, controlled environment, making it difficult to extrapolate the results to real-life CPR performance. Fourth, despite improvement in pCPR quality and retention of the skills, the results cannot be directly associated with patient-level outcomes. Fifth, this review only includes studies after 2005, therefore, it is not known if other important evidence exists prior to 2005. Finally, despite exhaustive attempts to locate every relevant resource, one study was identified but could not be retrieved.

## Conclusion

Brief and frequent pCPR retraining may result in a more appropriate skill retention and consequent high-quality performance. There is no strong evidence regarding the ideal retraining schedule however, it was demonstrated that, with as little as two minutes of refresher training every month, there is the potential to increase pCPR performance and retain the skills for longer.

## Funding

The authors declared that this research has not received any grant from any funding agency in the public, commercial or not-for-profit sectors.

## Conflict of Interests

The authors declare that there are no conflicts of interest.

## Appendix 1 Search terms

The search terms were drafted by the research team and revised by an experienced librarian.

TI (train* OR retrain* OR refresh* OR teach* OR educat* OR course* OR simulation OR update OR program*) OR AB (train* OR retrain* OR refresh* OR teach* OR educat* OR course* OR simulation OR update OR program*) AND

TI (“health*care professional*” OR “health*care worker*” OR physician* OR nurse* OR doctor*) OR AB (“health*care professional*” OR “health*care worker*” OR physician* OR nurse* OR doctor*)

TI (p*ediatric OR child* OR bab* OR infant) OR AB (p*ediatric OR child* OR bab* OR infant) AND

TI (resus* OR CPR OR “cardio*pulmonary resus*” OR “life support” OR BLS OR ALS OR PALS OR EPALS OR APLS OR PILS OR PHPLS OR ATLS OR “first aid” OR “chest compress*”) OR AB (resus* OR CPR OR “cardio*pulmonary resus*” OR “life support” OR BLS OR ALS OR PALS OR EPALS OR APLS OR PILS OR PHPLS OR ATLS OR “first aid” OR “chest compress*”) AND

TI (retention OR retain* N3 skill OR recall OR decay OR knowledge OR confidence) OR AB (retention OR retain* N3 skill OR recall OR decay OR knowledge OR confidence).
